# Exploring factors associated with the uneven utilization of telemedicine in Norway: a mixed methods study

**DOI:** 10.1186/s12911-017-0576-4

**Published:** 2017-12-28

**Authors:** H. Alami, M. P. Gagnon, R. Wootton, J. P. Fortin, P. Zanaboni

**Affiliations:** 1 Research Center on Healthcare and Services in Primary Care, Institute of Health and Social Services in Primary Care. Laval University (CERSSPL-UL). CIUSSS-CN, Pavillon Landry-Poulin, 2525, chemin de la canardiere, Quebec, QC G1J 0A4 Canada; 2 Research Centre of the CHU de Quebec-Universite Laval, Hopital St-François d’Assise, Edifice D, 45, rue Leclerc, Quebec, QC G1L 2G1 Canada; 3 Faculty of Nursing Science, Laval University. Pavillon Ferdinand-Vandry, 1050, avenue de la Medecine, Quebec, QC G1V 0A6 Canada; 4 Norwegian Centre for E-health Research, University Hospital of North Norway, P.O. Box 35, 9038 Tromso, Norway; 5 Faculty of Medicine, Laval University Pavillon Ferdinand-Vandry, 1050, avenue de la Medecine, Quebec, QC G1V 0A6 Canada

**Keywords:** Telemedicine, Implementation, Adoption, Utilization, Sustainability, Scaling-up, Health system

## Abstract

**Background:**

Norway has a long history of using telemedicine, especially for geographical reasons. Despite the availability of promising telemedicine applications and the implementation of national initiatives and policies, the sustainability and scaling-up of telemedicine in the health system is still far from accomplished. The main objective of this study was to explore and identify the multi-level (micro, meso and macro) factors affecting telemedicine utilization in Norway.

**Methods:**

We used a mixed methods approach. Data from a national registry were collected to analyze the use of outpatient visits and telemedicine contacts in Norway from 2009 to 2015. Interviews with key stakeholders at national, regional and local level helped complete and contextualize the data analysis and explore the main issues affecting the use of telemedicine by health authorities and hospitals. Relevant national documents were also used to support, contradict, contextualize or clarify information and data.

**Results:**

Telemedicine use in Norway from 2009 to 2015 remained very low, not exceeding 0.5% of total outpatient activity at regional level and 0.1% at national level. All four regions used telemedicine. Of the 29 hospitals, 24 used it at least once over the 7-year period. Telemedicine was not used regularly everywhere, with some hospitals using it sporadically. Telemedicine was mostly used in selected specialties, including rehabilitation, neurosurgery, skin and venereal diseases. Three major themes affecting implementation and utilization of telemedicine in Norway emerged: (i) governance and strategy; (ii) organizational and professional dimensions; (iii) economic and financial dimensions. For each theme, a number of factors and challenges faced at different health care levels were identified.

**Conclusions:**

This study allowed shedding light on multi-level and interdependent factors affecting utilization of telemedicine in Norway. The identification of the main implementation and utilization challenges might support decision makers and practitioners in the successful scaling-up of telemedicine. This work provides a knowledge base useful to other countries which intend to implement telemedicine or other digital health services into their healthcare systems.

**Electronic supplementary material:**

The online version of this article (10.1186/s12911-017-0576-4) contains supplementary material, which is available to authorized users.

## Background

Norway has a long history with use of information and communications technologies (ICTs) in health. With a dispersed population (5.3 million inhabitants on an area of about 400.000 sq. km) [1], a territory characterized by remote districts and a harsh climate, Norway had, since the beginning of the 1990s, sought innovative solutions to ensure equitable and continuous access to health services [2, 3]. The investment in ICTs has also been driven by the need for restructuring and rationalizing its public sector [4].

Norway has a tax-financed public healthcare system [5]. The State, as owner of the hospitals, is responsible for ensuring access to health services for the entire population, including those living in the most isolated and remote areas [6]. The organization of the healthcare system is semi-decentralized. At the central level, the Ministry of Health and Care Services develops and implements the national health policy by producing legislative frameworks and allocating resources. The Norwegian Directorate of Health, which reports to the Ministry, has a mandate to implement the Ministry’s policy and plans, and provide advice on strategies and legislation. ​On January 1st, 2016, the Norwegian Directorate of e-Health was also established with the intent to implement the national policy on e-health, determine the requisite standards, and administrate the use of e-health nationwide.

The country is divided into four regional health authorities (Northern, Central, Western, and South-Eastern Norway), which are state enterprises responsible for specialized healthcare services (i.e. hospital care) regionally [7, 8]. Hospitals are financed with a mix of activity-based funding (about 40%) and base funding (about 60%) [8, 9]. Private insurance remains low (about 2%) [8, 10]. Regions and hospitals have relative freedom to manage investments and to plan, organize and deliver services [11]. At the local level, municipalities are responsible for primary health services, including general practice, mental health care, nursing homes, preventive medicine and health promotion [12]. Municipalities are the patient’s first point of contact with the healthcare system via the general practitioner (GP), who can refer the patient to specialized care [8]. At the municipal level, health services are funded through national base funding, local taxes and out-of-pocket payments from citizens [13].

Telemedicine, defined as the "*use of communications networks for delivering healthcare services and medical education from one geographical location to another*" [14], can improve access to health services, particularly for people in remote areas [15, 16]. Norway introduced the first reimbursement for telemedicine in 1996 [17]. In 2010, the national health infrastructure Norwegian Health Network was implemented to secure exchange of medico-administrative data and information [3, 18]. This infrastructure is considered to be the backbone of telemedicine and e-health in Norway [19]. All hospitals, GPs and municipal health services have an electronic health record (EHR) [20]. Norway was also the first country to fully digitize radiology [21]. However, there is a gap between the implementation of telemedicine by the government and its actual use in regions and organizations [2]. Despite the early adoption of telemedicine, the use of these services remains still low and fragmented across the country [2, 14, 19, 22]. Norway faces the same problem of routinization and scaling-up of telemedicine services reported elsewhere in the world [23, 24].

The aims of the present study are: 1) to analyze and update telemedicine utilization data in Norway over the past 7 years, in continuity with previous reports on statewide adoption of telemedicine [2, 14], and 2) to explore and understand factors that may affect the use of telemedicine in the Norwegian healthcare system. The identification of some main implementation challenges might support decision makers and practitioners in the successful scaling-up of telemedicine. Finally, this work will provide a knowledge base useful to other countries which intend to implement telemedicine or other digital health services into their healthcare systems.

## Methods

We used a mixed methods approach [25], combining quantitative data collected from a national registry with qualitative information collected through interviews with key stakeholders, as well as additional documentation.

### Quantitative data

We collected data on the use of outpatient visits and telemedicine contacts in Norwegian hospitals from 2009 to 2015 from the Norwegian Patient Registry (NPR). The NPR is managed by the Norwegian Ministry of Health and Care Services and is used for planning, evaluation and funding of health services as well as for research purposes [14]. The NPR contains data related to inpatient and outpatient care provided by hospitals and based on activity-based funding. This means that only those activities, including telemedicine, which are reimbursed are included in the NPR. In Norway, a telemedicine activity is defined as the use of videoconferencing to conduct a consultation or examination, establish a diagnosis or provide treatment at distance [26]. There is currently no reimbursement for store-and-forward (not real-time) telemedicine.

### Qualitative data

We conducted semi-structured interviews with key stakeholders in telemedicine at national, regional and local levels in order to complete and contextualize the quantitative data, and explore the main issues affecting the use of telemedicine by regions and hospitals. The interview guide (Additional file 1) was developed on the basis of available literature on telemedicine in Norway, analysis of quantitative data, as well as from evaluations of telemedicine projects that some authors have conducted in the past. The interview guide included questions covering strategies, governance, organizational, professional, economic and financial issues, and was adapted to the type of respondents. Potential respondents were identified primarily through the contact network of the Norwegian Centre for E-health Research by contacting a representative in each target group (national, regional and local) and obtaining suggestions of other relevant persons with extensive knowledge and experience on telemedicine in Norway, following a snowballing technique [27]. Internet searches were also conducted to identify other key people in participating organizations, particularly via organizational and Government documents or reports dealing with e-health. A list of nearly 30 potential respondents was identified. In total, 9 interviews were conducted with key stakeholders in hospitals, regional and national health authorities and universities (Table 1). Most of respondents had both a clinical and managerial profile.

**Table 1:** Summary of qualitative data collected through interviews and documents

Qualitative data	Number
Interviews	
Hospitals	5
Regional and national health authorities	3
Universities	1
Documents	
Government or learned societies reports and documents	16
Articles	17
Academic reports and thesis	4
Total	37

Relevant national documents on telemedicine (e.g. reports, evaluations, articles) were also used to reconstruct the sequence of key events and situate them in their context, while highlighting the critical decisions and the reasons why they were made, but also to support, contradict, contextualize or clarify some information obtained (Table 1).

### Data analysis

Quantitative data were stratified by region, hospital, clinical specialty, and year. Relative use of telemedicine was expressed as the proportion of telemedicine contacts over the number of outpatient visits. Adoption was expressed as percentage of the number of users over the potential users [14]. The remoteness of each region was measured with two indexes which enable to estimate the peripherality of Norwegian municipalities [14, 28]: 1) the population index (scored 0–10), which represents the population density of a municipality; and 2) the centrality index (scored 0–20), which describes the geographic location of a municipality based of the largest urban centre that can be reached within a given travel time. For each region, the indexes were calculated as the median of the values of all municipalities belonging to that region. High values correspond to less isolated and more populated areas, respectively [2].

Interviews were recorded and transcribed. Then, the transcriptions and observation notes were subjected to a “pragmatic” thematic analysis of the content [29], conducted by HA and PZ to identify themes. Having not taken a particular conceptual framework for the study, we proceeded in an inductive-deductive way. This flexibility has made it possible to start from some dimensions that were regularly reported (e.g. professional, organizational, and economic) in the literature [30], while leaving the perspective open to other potential elements that could emerge from the data. Themes were discussed and subsequently validated.

The results from the quantitative data, the findings from the interviews as well as those from the documentation were then analyzed by data triangulation [31]. By regularly returning to the primary sources of data, this approach allowed comparing multiple sources, verify and identify convergences or divergences, complete and reformulate observations and findings [32, 33].

This study did not require the participation of human subjects. The information collected from the NRP and the semi-structured interviews was anonymous and no identifiable information or data related to individuals were collected or are accessible. According to the Norwegian Health Research Act and the Personal Data Act, ethics approval was therefore not necessary.

## Results

### Adoption and utilization of telemedicine in Norway

**Fig. 1:**
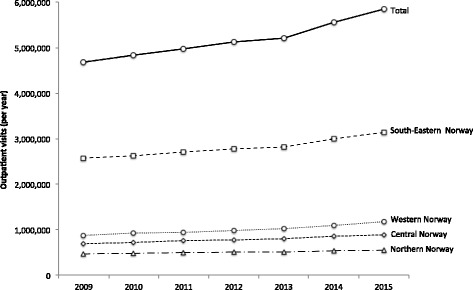
Outpatient visits in the period 2009–2015 in Norway and in the four health regions

**Fig. 2:**
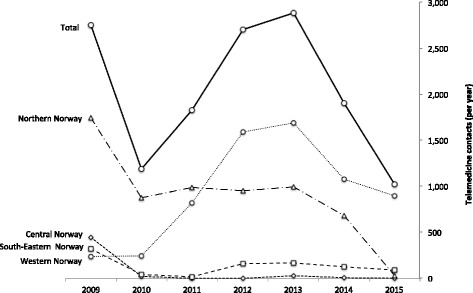
Telemedicine contacts in the period 2009–2015 in Norway and in the four health regions

All four regions used telemedicine to some extent (Table 2). However, its use remained very low, not exceeding 0.5% of total outpatient activity in each region, and 0.1% of the total outpatient activity across the country. Telemedicine activity was not characterized by the same steady growth experienced by outpatient visits (Fig. 1). Telemedicine activity declined first in 2010, except for Western Norway (Fig. 2). Western Norway experienced then a significant increase in telemedicine activity, reaching 0.16% in 2013 (0.03% in 2009). Telemedicine activity in the other regions remained generally unchanged over this period, with South-Eastern Norway and Central Norway characterized by a lower activity than Northern Norway. From 2013, telemedicine activity declined significantly across all regions.

**Table 2:** Outpatient visits and telemedicine contacts in the period 2009–2015 in the four health regions in Norway

Health region	Centrality (0–20)^a^	Population (0–10)^b^	Outpatient visits (2009)	Outpatient visits (2010)	Outpatient visits (2011)	Outpatient visits (2012)	Outpatient visits (2013)	Outpatient visits (2014)	Outpatient visits (2015)	Telemedicine contacts (2009)	Telemedicine contacts (2010)	Telemedicine contacts (2011)	Telemedicine contacts (2012)	Telemedicine contacts (2013)	Telemedicine contacts (2014)	Telemedicine contacts (2015)
Western Norway	10	0.80	879,911	930,840	947,303	994,769	1,027,463	1,103,811	1,178,069	240 (0.03%)	246 (0.03%)	821 (0.09%)	1586 (0.16%)	1686 (0.16%)	1077 (0.10%)	894 (0.08%)
Central Norway	11	0.50	695,162	724,617	763,467	784,757	804,753	864,541	892,276	448 (0.06%)	23 (0.00%)	1 (0.00%)	0 (0.00%)	32 (0.00%)	11 (0.00%)	0 (0.00%)
Northern Norway	4	0.20	470,078	484,151	502,839	515,029	514,316	539,145	552,830	1739 (0.37%)	876 (0.18%)	986 (0.20%)	955 (0.19%)	991 (0.19%)	680 (0.13%)	32 (0.01%)
South-Eastern Norway	14	1.30	2,573,532	2,625,076	2,711,593	2,783,087	2,819,066	2,994,430	3,134,280	318 (0.01%)	41 (0.00%)	19 (0.00%)	159 (0.01%)	170 (0.01%)	130 (0.00%)	95 (0.00%)
Private sector	–	–	57,563	61,065	35,424	34,320	37,590	50,582	75,336	0 (0.00%)	0 (0.00%)	0 (0.00%)	0 (0.00%)	0 (0.00%)	0 (0.00%)	0 (0.00%)
Total	12	0.6	4,676,246	4,825,749	4,960,626	5,111,962	5,203,188	5,552,509	5,832,791	2745 (0.06%)	1186 (0.02%)	1827 (0.04%)	2700 (0.05%)	2879 (0.06%)	1898 (0.03%)	1021 (0.02%)

^a^Low values correspond to more isolated areas

^b^Low values correspond to less populated areas

Overall, telemedicine activity was higher in regions characterized by a lower centrality (Table 2). Western Norway was the region using most telemedicine across the whole period. Northern Norway, region characterized by a very low population density (score: 0.20) and peripherality (score: 4), reported consistently use of telemedicine with the exception of a decrease in 2015.

At the hospital level, 24 hospitals (out of 29, excluding the private sector) used telemedicine at least once over the period 2009–2015 (Table 3). However, telemedicine was not used evenly across hospitals. Only four hospitals used telemedicine continuously (*Universitetssykehuset i Nord-Norge*, *Helse Finnmark* and *Nordlandssykehuset HF* in Northern Norway, and *Helse Stavanger* in Western Norway). There were 13 hospitals using telemedicine in 2009, and the same number in 2015. However, they were different hospitals: some have stopped using telemedicine, while others adopted it later.

**Table 3:** Outpatient visits and telemedicine consultations in the period 2009–2015 in Norwegian hospitals

Hospital	Outpatient visits (2009)	Outpatient visits (2010)	Outpatient visits (2011)	Outpatient visits (2012)	Outpatient visits (2013)	Outpatient visits (2014)	Outpatient visits (2015)	Telemedicine contacts (2009)	Telemedicine contacts (2010)	Telemedicine contacts (2011)	Telemedicine contacts (2012)	Telemedicine contacts (2013)	Telemedicine contacts (2014)	Telemedicine contacts (2015)
Western Norway	879,911	930,840	947,303	994,769	1027,463	1,103,811	1,178,069	240 (0.03%)	246 (0.03%)	821 (0.09%)	1586 (0.16%)	1686 (0.16%)	1077 (0.10%)	894 (0.08%)
Hospitalet Betanien (Hordaland)	1675	2059	2097	2104	2192	1191	2840	0 (0.00%)	0 (0.00%)	0 (0.00%)	0 (0.00%)	0 (0.00%)	0 (0.00%)	0 (0.00%)
Haugesund san.for. Revmatismesh	21,914	21,066	18,082	24,333	23,915	27,803	28,129	2 (0.01%)	0 (0.00%)	0 (0.00%)	0 (0.00%)	0 (0.00%)	0 (0.00%)	0 (0.00%)
Helse Stavanger	236,601	274,315	268,052	279,797	289,860	304,372	327,738	124 (0.05%)	201 (0.07%)	806 (0.30%)	1583 (0.57%)	1684 (0.58%)	939 (0.31%)	203 (0.06%)
Helse Fonna	115,059	118,160	117,049	121,380	122,381	131,643	137,410	103 (0.09%)	41 (0.03%)	13 (0.01%)	1 (0.00%)	0 (0.00%)	0 (0.00%)	4 (0.00%)
Helse Bergen	376,996	388,058	409,798	432,519	448,597	487,688	520,773	0 (0.00%)	1 (0.00%)	2 (0.00%)	2 (0.00%)	0 (0.00%)	2 (0.00%)	7 (0.00%)
Helse Førde	110,630	109,995	112,956	114,554	119,052	127,160	133,408	11 (0.01%)	2 (0.00%)	0 (0.00%)	0 (0.00%)	2 (0.00%)	135 (0.11%)	680 (0.51%)
Haraldsplass diakonale sykehus	17,036	17,187	19,269	20,082	21,466	23,954	27,771	0 (0.00%)	1 (0.01%)	0 (0.00%)	0 (0.00%)	0 (0.00%)	1 (0.00%)	0 (0.00%)
Central Norway	695,162	724,617	763,467	784,757	804,753	864,541	892,276	448 (0.06%)	23 (0.00%)	1 (0.00%)	0 (0.00%)	32 (0.00%)	11 (0.00%)	0 (0.00%)
St. Olavs Hospital	327,390	350,338	368,701	382,669	393,556	420,927	429,956	448 (0.14%)	23 (0.01%)	1 (0.00%)	0 (0.00%)	10 (0.00%)	2 (0.00%)	0 (0.00%)
Helse Nord-Trøndelag	100,797	99,562	109,382	109,110	112,597	124,983	130,311	0 (0.00%)	0 (0.00%)	0 (0.00%)	0 (0.00%)	22 (0.02%)	9 (0.01%)	0 (0.00%)
Helse Møre og Romsdal	266,975	274,717	285,384	292,978	298,600	318,631	332,009	0 (0.00%)	0 (0.00%)	0 (0.00%)	0 (0.00%)	0 (0.00%)	0 (0.00%)	0 (0.00%)
Northern Norway	470,078	484,151	502,839	515,029	514,316	539,145	552,830	1739 (0.37%)	876 (0.18%)	986 (0.20%)	955 (0.19%)	991 (0.19%)	680 (0.13%)	32 (0.01%)
Helse Finnmark	55,048	54,132	55,108	59,607	59,092	63,866	67,269	14 (0.03%)	33 (0.06%)	39 (0.07%)	105 (0.18%)	76 (0.13%)	55 (0.09%)	1 (0.00%)
Universitetssykehuset i Nord-Norge	214,538	227,831	235,486	238,232	241,248	247,151	252,586	1325 (0.62%)	780 (0.34%)	848 (0.36%)	558 (0.23%)	778 (0.32%)	520 (0.21%)	31 (0.01%)
Nordlandssykehuset	122,723	126,532	130,953	132,566	133,766	143,734	144,424	147 (0.12%)	63 (0.05%)	99 (0.08%)	292 (0.22%)	137 (0.10%)	105 (0.07%)	0 (0.00%)
Helgelandssykehuset	77,769	75,656	81,292	84,624	80,210	84,394	88,551	253 (0.33%)	0 (0.00%)	0 (0.00%)	0 (0.00%)	0 (0.00%)	0 (0.00%)	0 (0.00%)
Private sector	57,563	61,065	35,424	34,320	37,590	50,582	75,336	0 (0.00%)	0 (0.00%)	0 (0.00%)	0 (0.00%)	0 (0.00%)	0 (0.00%)	0 (0.00%)
South-Eastern Norway	2,573,532	2,625,076	2,711,593	2,783,087	2,819,066	2,994,430	313,4280	318 (0.01%)	41 (0.00%)	19 (0.00%)	159 (0.01%)	170 (0.01%)	130 (0.00%)	95 (0.00%)
Betanien hospital (Telemark)	14,868	16,983	18,760	19,815	19,642	22,212	24,025	0 (0.00%)	0 (0.00%)	0 (0.00%)	0 (0.00%)	1 (0.01%)	0 (0.00%)	0 (0.00%)
Sunnaas sykehus	2691	3922	3598	3285	4388	4791	4740	0 (0.00%)	4 (0.10%)	5 (0.14%)	132 (4.02%)	154 (3.51%)	106 (2.21%)	67 (1.41%)
Vestre Viken	287,427	277,960	296,535	306,315	326,293	353,262	388,393	0 (0.00%)	3 (0.00%)	1 (0.00%)	0 (0.00%)	1 (0.00%)	5 (0.00%)	2 (0.00%)
Lovisenberg	43,071	45,088	52,065	53,489	57,058	58,513	62,055	10 (0.02%)	0 (0.00%)	0 (0.00%)	0 (0.00%)	0 (0.00%)	0 (0.00%)	0 (0.00%)
Martina Hansens hospital	22,934	22,964	25,021	29,528	29,568	31,409	32,084	0 (0.00%)	0 (0.00%)	0 (0.00%)	0 (0.00%)	0 (0.00%)	0 (0.00%)	0 (0.00%)
Revmatismesykehuset Lillehammer	10,701	10,803	12,351	13,916	13,960	13,117	13,718	0 (0.00%)	0 (0.00%)	0 (0.00%)	0 (0.00%)	0 (0.00%)	0 (0.00%)	0 (0.00%)
Diakonhjemmet	51,898	55,768	62,841	65,258	66,825	69,953	72,919	0 (0.00%)	0 (0.00%)	0 (0.00%)	0 (0.00%)	0 (0.00%)	0 (0.00%)	0 (0.00%)
Akershus universitetssykehus	175,830	185,536	233,530	254,194	248,798	281,331	297,114	0 (0.00%)	0 (0.00%)	0 (0.00%)	0 (0.00%)	0 (0.00%)	0 (0.00%)	3 (0.00%)
Sykehuset Innlandet	317,634	320,325	327,537	335,019	341,459	367,332	378,203	97 (0.03%)	14 (0.00%)	1 (0.00%)	4 (0.00%)	1 (0.00%)	0 (0.00%)	0 (0.00%)
Sykehuset Østfold	200,674	195,314	196,563	205,507	212,247	225,557	240,744	137 (0.07%)	5 (0.00%)	2 (0.00%)	3 (0.00%)	0 (0.00%)	0 (0.00%)	1 (0.00%)
Sørlandet sykehus	267,781	271,263	279,041	292,567	298,291	310,754	320,442	74 (0.03%)	15 (0.01%)	8 (0.00%)	18 (0.01%)	13 (0.00%)	13 (0.00%)	7 (0.00%)
Sykehuset i Vestfold	196,826	195,674	205,989	215,857	213,254	227,522	236,018	0 (0.00%)	0 (0.00%)	2 (0.00%)	1 (0.00%)	0 (0.00%)	1 (0.00%)	0 (0.00%)
Sykehuset Telemark	155,306	164,000	169,598	173,197	154,658	173,307	178,888	0 (0.00%)	0 (0.00%)	0 (0.00%)	0 (0.00%)	0 (0.00%)	3 (0.00%)	2 (0.00%)
Oslo kommunale legevakt	–	–	–	–	12	160	20	–	–	–	–	0 (0.00%)	0 (0.00%)	0 (0.00%)
Oslo universitetssykehus	825,891	859,476	828,164	815,140	832,613	855,210	884,917	0 (0.00%)	0 (0.00%)	0 (0.00%)	1 (0.00%)	0 (0.00%)	2 (0.00%)	13 (0.00%)
Total	4,676,246	4,825,749	4,960,626	5,111,962	5,203,188	5,552,509	5,832,791	2745 (0.06%)	1186 (0.02%)	1827 (0.04%)	2700 (0.05%)	2879 (0.06%)	1898 (0.03%)	1021 (0.02%)

Ten hospitals conducted more than 100 consultations for at least 1 year from 2009 to 2015. Only two hospitals conducted more than 1000 consultations for at least 1 year. These two hospitals (*Universitetssykehuset i Nord-Norge* and *Helse Stavanger*) are located in the two regions which are most active in telemedicine, Northern Norway and Western Norway. However, telemedicine activities were often oriented towards one or two specialties: in *Universitetssykehuset i Nord-Norge*, more than 95% of the total telemedicine activity occurred in neurosurgery and eye diseases; in *Helse Stavenger*, physical medicine-rehabilitation alone had more than 95% of the total telemedicine activity. Other hospitals in Norway had a very low telemedicine activity. For example, *Oslo Universitetssykehus*, the hospital with the largest outpatient activity in Norway, had no telemedicine activity in 2009 and only 13 telemedicine consultations in 2015.

At the clinical level, telemedicine was used in 29 out of 45 different clinical specialties in the hospitals between 2009 and 2015 (Additional file 2). Only 16 clinical specialties recorded at least one telemedicine consultation in 2015, while telemedicine was used in 27 specialties in 2009. The clinical specialties that account for the larger part of telemedicine activities in hospitals were: physical medicine-rehabilitation, neurosurgery, skin and venereal diseases, with more than 500 consultations for at least 1 year.

### Exploratory analysis of the factors affecting telemedicine utilization

The use of telemedicine in Norway remained low. In addition, utilization was fragmented among organizations and regions, and was concentrated mainly in selected specialties. Three major themes regarding factors affecting use of telemedicine in Norway emerged from the exploratory analysis of the qualitative data collected from interviews and documents: (i) governance and strategy; (ii) organizational and professional dimensions; (iii) economic and financial dimensions. A number of factors are described for each theme, with quotes from the interviewees marked with the respondent’s number (e.g. R1, R2).

#### Governance and strategy

According to policy makers, Norway began after 2013 a significant shift towards a more global e-health strategy, which might partially explain the general decline and the low use of telemedicine in the country in the recent years.

*« (…) In the beginning (1990s and 2000s) we had a strategy for telemedicine. But I feel that telemedicine is not in the agenda anymore. Now, we have a national strategy in e-health, and telemedicine is a part of e-health (…) ». (R1).*

This shift was explained by the fact that several telemedicine projects were conducted and different technologies tested over the years. However, telemedicine is still not widely integrated in the routine provision of health services. Respondents recognized that the proliferation of autonomous and fragmented local initiatives resulted in difficulties in the integration, harmonization and coordination of telemedicine activity. Moreover, some respondents believe that previous national strategies were mainly focused on messaging services and electronic exchange, while less attention was paid towards telemedicine services, especially videoconferencing [34]. This may also explain the low use of telemedicine in Norway. Telemedicine could no longer be considered separately from other national e-health initiatives. As a consequence, a comprehensive and integrated strategy for e-health has recently been implemented, with a need for systems of electronic exchange and stable infrastructure to ensure appropriate access to information.

At the regional level, respondents acknowledged that governance of telemedicine is difficult. Despite the recent shift towards e-health, infrastructure, expertise, and predisposition toward telemedicine differ across the four regions, with a consequent challenge for the implementation of the national strategy. This can explain the differences observed between Northern Norway and Western Norway in terms of telemedicine utilization. Moreover, there is still a lack of coordination between healthcare levels, especially between municipalities and hospitals. This fragmentation could be explained by institutional boundaries. Hospitals and municipalities belong to regional and local level, respectively, each involving different administrative, professional and political cultures, as well as different funding mechanisms. There is also a great variability in terms of expertise, needs and financial resources available at each level.

«*Our organization is eager to get municipalities to use telemedicine, and some of them use it often. But there is much work to be done before all the 26 municipalities use telemedicine towards our specialists and services (...). There is an important need for information, leadership and change management strategies to support them (…). Most municipalities don’t have enough funds and skills needed to implement telemedicine services». (R2).*

The diversity of governance levels and stakeholders makes the situation even more complex. Telemedicine requires a variety of technologies, services and procedures with fragmented ownership. As a consequence, responsibility (e.g. maintenance, updating, traceability, data management, authentication and security) of this set represents an unresolved issue. Fragmentation reduces exchange and sharing of information between organizations (hospitals) and levels (regions and municipalities).

« *(…) One of the biggest problems is the integration of information. Information can be shared between systems [*e.g. *hospital-hospital, hospital-municipality] (...). But if you want to integrate systems, at the moment it’s far too complex (…). Expertly, we need to simplify the set-up, that it’s not easy too (…). Moreover, you should know that in Norway the information is recorded and classified by structure and not by the patient, which can cause a loss of information (…). So sharing and continuity of information is still a big problem (…). Paradoxically, we have more sharing of information between patients and organizations than between organizations, because patients have legal rights for access to their information (…) ». (R3).*

Finally, the Coordination reform [35], which aimed at strengthening collaboration between primary care services and specialized healthcare services, had a significant impact on the decline in telemedicine utilization, especially after 2009. This reform introduced many changes in the division of work and responsibilities between organizations. For example, telemedicine services were unable to be operationalized for the lack of a clear division of responsibilities between stakeholders involved [3]. Additional challenges emerged also from the appearance of the first difficulties related to the implementation of the coordination agreements. Stakeholders were not sufficiently prepared on the complexity of this reform, particularly in terms of sharing of responsibilities and funding. The availability of regulatory and legal instruments to clarify responsibilities, including contractual arrangements, procedures and guidelines, was missing. Respondents also reported that the current national guidelines and standards are not detailed and do not fully meet the needs of organizations.

#### Organizational and professional dimensions

Telemedicine in Norway has been implemented mostly as projects, while a telemedicine strategy is still lacking in most organizations. Many projects were the result of leaders’ initiatives (managers or doctors), and only some hospitals included telemedicine in their medium and long-term organizational strategies. It was also reported that the success and continuity of these projects depend on the willingness of clinicians to use telemedicine. Some respondents stated that it takes time to convince people to use telemedicine and provide training and support. The high turnover of clinicians was highlighted as an additional barrier.

*« (…) By the organizational guidelines, doctors are obliged to use telemedicine, but in practice they have to be convinced that telemedicine is a good tool. Therefore, it is very important that doctors who use telemedicine can be ambassadors towards other colleagues (…). New clinicians get help to choose the right equipment for their use, they are educated in how to use the equipment and are followed up when needed (…) ». (R5).*

Telemedicine was also seen as an organizational development issue. According to respondents, telemedicine requires a long process of learning and adaptation, both individually and collectively, to ensure successful integration into an organization. This might include changes in processes, practices, cultures, communication, and division of work. Time, capacity and resources required may differ depending on the maturity of an organization and its ability to integrate changes and initiate restructuring. In most organization, such conditions are not yet sufficiently satisfied.

*« (…) It’s still very hard to use telemedicine systems. It’s difficult to organize, especially if you don’t have so much time and many patients (…). Then, it’s easier to send them to the hospital than to organize a telemedicine consultation and keep responsibility for follow-up (…)* ». *(R5).*

Telemedicine projects have been mostly supported by internal funding within organizations, and initiatives often promoted by local champions. This makes the sustainability and scaling-up of services difficult once the pilot phase is completed. Only a few projects could be considered to be integrated into the routine practice (e.g. eye diseases, neurosurgery, physical medicine-rehabilitation). However, due to the lack of recurrent funding, telemedicine services risk to be discontinued as the organization alone cannot bear the costs. External financing towards telemedicine comes mainly from regional or national programs supporting research projects and innovations. According to some respondents, this funding is very hard to obtain and requires several efforts for organizations. Moreover, funding is often oriented towards specific applications. This explains the higher utilization of telemedicine in selected clinical specialties. The presence of such non-recurring funding from research and innovation initiatives, which expires after the project end, might partially justify the decline of telemedicine activity in certain periods.

« *(…) we hope that the project financing will become a financing activity. Or else, we are forced to look to other funds. If we don’t, we will have to close down the project (…)* ». *(R4).*

Finally, small hospitals are more likely to refuse using telemedicine. As telemedicine decreases the need for physical presence (even part-time) of clinicians, small hospitals might face even more difficulties in retaining or recruiting clinicians on site.

#### Economic and financial dimensions

There is a consensus that the current compensation model represents a major obstacle to the use of telemedicine, especially for services between primary care (municipalities) and specialized care (hospitals). Reimbursement is currently recognized solely to teleconsultations occurring via videoconferencing between the patient and at least one health professional, of whom at least one is a doctor, from two different physical locations. The reimbursement, equal to that of a face-to-face visit, is provided to the specialist doctor only, while there is not any reimbursement for GPs or other health professionals involved in a teleconsultation. Moreover, conducting a teleconsultation implies additional work for the GP (e.g. planning and organizing appointments with the specialist). As a consequence, GPs often decide to refer the patient to the hospital rather than using telemedicine. A lack of reimbursement applies also to telemedicine activities where only nurses and other healthcare professionals are normally involved in the service provision. The current funding model represents a major obstacle to a widespread use of telemedicine and interprofessional collaboration.

« *(…) To be accounted in financing activity, there must be a doctor who consults a patient. But in one of our projects it is the nurse who is in contact with the patient. The activity-based funding does not take into account this aspect. We hope that this will change (…)* ». *(R3).*

Another important factor reported was related to the redistribution of the savings among organizations, which could represent a disincentive to use telemedicine. For example, hospitals providing a specialist teleconsultation perform this activity without taking over the patient physically. At the same time, health authorities can save on travel expenses. The reimbursement of these costs in Northern Norway accounts for $1.4 billion per year, about 10% of its health budget [34, 36].

« *(…) We had an agreement with some doctors to visit some small remote cities and hospitals. These visits allowed us to not transfer patients (…). When we started with telemedicine, these specialists did not need to travel to visit patients in these small cities, as they have the possibility to do it at distance (…). But one of the problems was that those doctors received very good incomes for traveling to these cities. Then, with telemedicine they didn’t get these supplement incomes any longer. So, some doctors are more interested to travel to get these incomes. Then, they don’t use telemedicine (…) ». (R1).*

Finally, hospitals and municipalities have different budget and funding models, as well as distinct administrative operations. Telemedicine implies a redistribution of tasks, workload, costs and savings between different parties which have their own autonomy and funding sources. The establishment of co-financing and co-responsibility models is a challenge, especially for the management of investments in equipment and services, but also for sharing of savings and responsibilities.

*« (…) The problem is that there is no connection between different levels or types of financing. You can see for transport, time used, technology, human resources,* etc. *Each one has a different source of financing. So, it’s not possible to see all this together (…). You have responsibilities, costs and benefits to share with many levels and stakeholders. This is a very big problem for telemedicine in Norway (…). So, it’s very important for leaders and institutions to try to study the whole economic situation. This is the most important issue (…) ». (R1).*

## Discussion

This study, based on a mixed methods approach, allowed identifying a number of factors and challenges faced at different health care levels which must be addressed to support to the successful integration of telemedicine into the routine of health services in Norway.

### Statewide adoption and use of telemedicine

Telemedicine is not “new” for both hospitals and regions in Norway. At least 24 hospitals (out of a total of 29) and the four regions used telemedicine at one time or another over the period 2009–2015. However, telemedicine has been mainly used to provide access to certain health services for populations living in remote areas. Hospitals that are “heavy users” are located in regions with lower centrality, especially in Northern Norway. Despite Norway has a nationwide network where telemedicine is used in several hospitals and specialties, utilization remains overall low and fragmented. Indeed, telemedicine use in hospitals is focused in selected specialties, especially physical medicine/rehabilitation and neurosurgery. Such situation can be explained as much by the leadership of some champions in these specialties, the lack of organizational strategies at hospital and regional levels as well as recurring funding.

Thus, in view of this weak and fragmented use across the country, exploitation of the full potential of telemedicine compared with the existing need (identified or not) is still far from being reached, especially considering the growth in outpatient visits in Norway (+ 24.7% between 2009 and 2015) (Table 2).

The general context remains relatively favorable to a larger use of telemedicine at national level. However, to achieve this objective, certain issues and questions identified in this work should be addressed. Indeed, evidence of efficacy or cost-effectiveness (theoretical or real), although essential, of telemedicine alone is insufficient to ensure its successful integration in the healthcare system.

#### Strategy, governance, organization and coordination of telemedicine

Norway was the first country to implement a reimbursement for telemedicine in 1996, which had a positive impact on utilization [14]. Moreover, several national action plans have been implemented over the years to support technological infrastructure and regulation: «More health for each bIT» (2001–2003), «Say@!» (HOD, 2001), «Te@mwork (2004–2007), «Interaction 2.0» [37], and «One Citizen–One Record» (2012–2013) [34]. Add to this, the «Coordination reform» (2009) focused on ICTs as a leverage to strengthen coordination of services between healthcare levels [21, 35] and to offer the best services to the population with emphasis on collaboration and exchange of information [21, 35]. However, these national plans and strategies were mainly focused on messaging services and electronic exchange, and less on telemedicine services, especially videoconferencing [34], whereas it should be an integral part of these strategies.

There is still a lack of coordination across national, regional, and local level. Telemedicine initiatives are often characterized by a multitude of actors (e.g. hospitals and municipalities) who have different political, administrative, fiscal, legal, clinical and technological models, as well as different objectives, expectations, and perceptions [19]. Coordination around national plans is considered as an important prerequisite for successful implementation and integration of telemedicine [19].

The fragmentation characterizing the healthcare sector is another major obstacle to the development of an integrated nationwide telemedicine network. Issues related to management, storage, security, traceability and exchange of medico-administrative information between organizations are not yet fully resolved [19, 38]. Add to this that telemedicine involves services with fragmented stakeholders and owners, with consequent problems regarding shared responsibility of security, maintenance and use of technology.

Institutions have different infrastructures and processes, which are not necessarily interoperable. This adds complexity to the challenge of harmonizing and aligning different systems. Implementation of telemedicine requires adapting the solutions to the capacity of institutions to incorporate organizational changes, considering their needs, and making available the necessary resources and skills. Moreover, to build an integrated telemedicine network, it is necessary to coordinate institutional strategic plans and align them with national strategies and policies, while ensuring quality and safety for patients. Certification, quality, interoperability and security standards are relevant issues to be addressed [36, 39–41].

As a strategic innovation, telemedicine may experience problems of incompatibility or conflict with established governance models [42]. For instance, the decision to integrate telemedicine into the healthcare system is under government control, while the decision to implement it in practice is under the responsibility of the organizations [43], which have their own political, organizational, administrative and professional autonomy [40]. This requires finding a synergy between different levels of governance, balancing the national vision for e-health with the flexibility necessary for organizations and professionals to make choices tailored to their needs. Such a balance would also allow organizations to develop new skills and organizational routines and, as a consequence, to innovate [44, 45]. Digital transformation is less a matter of technology than strategy, vision and the development of new abilities and skills to work, collaborate and to experiment [46].

Finally, implementation of telemedicine may redefine the nature of the activities that healthcare professionals have to perform. Resistance to change can occur when actors do not perceive clinical, professional or financial benefits (*relative benefits*) or when they feel that they don’t have the necessary skills or resources (*visibility and complexity*). This creates a situation of uncertainty [47–50]. Change management strategies, communication and support, are central to the successful adoption and integration of innovations into organizations [51]. A collaborative, participatory and iterative approach involving all stakeholders can allow to better identify needs and priorities, thus translating them into clear and realistic objectives [50, 52].

#### Funding of telemedicine

Another challenge is the development of an economic model that allows sharing costs and benefits among stakeholders. Currently, reimbursement for telemedicine in Norway in specialized care is limited to teleconsultations occurring via videoconferencing deemed to replace physical consultations, and it is provided only to specialist doctors at the hospital. A number of store-and-forward telemedicine services have been used in Norwegian hospitals, such as teledermatology offered by means of still image referral, telepathology and telecardiology for home monitoring of patients with implantable cardioverter defibrillator and pacemaker [14, 19]. Despite Norway introduced the first reimbursement for telemedicine in 1996 for use of both store-and-forward and videoconferencing solutions in specialized care, reimbursement for store-and-forward telemedicine was discontinued in 2008 [2, 17]. Probably as a consequence, most services in routine use today occur via videoconferencing. Moreover, there are no financial incentives for GPs or other health professionals. Such economic constraints are often justified by the fear of increasing healthcare expenditures. However, the costs for the provision of telemedicine represent generally a small part of the total health expenditure [53]. It is therefore necessary to review the reimbursement policy to support routine use of telemedicine, interprofessional collaboration and information exchange. Reimbursement of services provided by specialists practising in private hospitals should also be explored [19]. The recent strategy for e-health in Norway seems to set the basis for a major shift towards nationwide large-scale use of digital health services to citizens and health professionals, including e-health and telemedicine in both specialized healthcare and primary care. An example is the recent introduction of a specific reimbursement for the conduction of (store-and-forward) e-consultations by GPs for patient follow-up. This is the first reimbursement policy to be introduced in Norway in primary care. Due to the current changes in strategy and policies for remuneration, it is important to monitor future revisions of reimbursement strategies and evaluate their impact on telemedicine adoption and utilization. The «EU eHealth Action Plan 2012–2020» should also contribute to drive changes in this direction, particularly in the light of recommendations aimed at implementing coherent policies and strategies to develop citizen/patient-centred care and services [54]. Moreover, new modes of professional health practice (e.g. use of smartphone or tablet to perform a medical consultation or follow-up by a health professional, mobility, work from home) and new forms of “network-organizations” should be considered in the future remuneration and financing mechanisms.

In addition to reimbursement policies, it is important to develop mechanisms promoting the redistribution of benefits (e.g. due to avoidance of travel) among all stakeholders. The lack of a suitable financing system contributes to a delay in the implementation of the restructuring changes necessary to achieve better integration of services between different levels. That said, the financing chain should take into account economic integration between front-line services (municipalities) and specialized services (hospital); so that this funding is adapted to the path of patients in the healthcare system and not the reverse [55]. In the same vein, investments in infrastructure and equipment must be covered by an economic framework of common expenses (e.g. between municipalities and hospitals) [19].

Otherwise, telemedicine projects have been mainly supported by internal funds within organizations, or through national or regional funding for research and innovation projects. Such mechanisms have a limited duration. Indeed, innovation involves slow organizational developments and transformations that require substantial investments, expensive sometimes, over long periods [56]. The development of long-term financing mechanisms, with flexible and suitable funding strategies, is important for the sustainability and scaling-up of telemedicine [45].

In sum, these elements echo the findings of other studies that recognize that the complexity characterizing telemedicine is often underestimated [57]. In this vein, it is reported that the factors of success and sustainability are multidimensional, ranging from technological and infrastructure issues, change management, professional practices, regulation, business and economic models, and organizational issues; this knowing that telemedicine allows to interconnect organizations that can have different practical models of governance, cultures, and organizational objectives, sometimes even antagonistic (e.g. performance criteria, etc.). The question of “e-readiness” (technological, professional, organizational, economic, political and societal) becomes thus unavoidable [57–59].

### International comparison perspective

The national policies, types of governance, strategies and regulations are now recognized as having a fundamental role in the success and scaling-up of telemedicine in health systems [23, 56, 60, 61]. Comparative studies between countries could be source of essential lessons, in particular by creating an incentive for these countries to conduct own monitoring programs and develop networks of experts who share their experiences, thus generating favorable contexts to the translation of knowledge into action. We observed that there are only relatively few studies that have analyzed the use and integration of telemedicine at the health system level.

There are some experiences that show interesting results in terms of sustainability and scaling-up of telemedicine services at the health systems level, namely, among others: the Ontario Telemedicine Network (Canada) [16, 62], the Alaska Federal Health Care Access Network [63, 64], the Veterans Health Administration Telehealth Network [65], and the Brazilian telehealth network [66]. These services should be better studied with a holistic approach to help better identify multilevel factors that affect (positively or negatively) the scaling-up and integration of telemedicine in health systems. Indeed, based on the fact that the evidence available is promising but incomplete, and sometimes incoherent [67, 68], more comparison works at the country level are needed [14, 68], especially on: strategies, governance models (centralized, decentralized, etc.), regulations, financing and reimbursement models, business models and the role of companies, the place of insurances (public and private), organizational models and service architecture. These comparisons could provide additional light on the health system challenges [14, 68]. This would imply going beyond studies of single projects, often initiated in silos (organizational or by specialty), usually in academic circles or in contexts of individual excellence, and without an overall vision or clear alignment on national policies and the organization of health services [69].

However, the lack of data collection and recording standards over several years, added to the cultural, political and health system context of each country, makes information sharing and international comparisons difficult. It would therefore be relevant to collect data on the use of telemedicine at health system level not only for billing and accounting purposes, but also to facilitate research and evaluation of services [16, 60].

Previous studies have explored the factors (micro, meso, macro) that impact on and influence telemedicine adoption and utilization in specific jurisdictictions. As such, several theoretical and conceptual frameworks have been proposed to better understand these conditions [57, 70–76]. These frameworks are generally agreed on several dimensions (socio-political, economic, regulatory, organizational, professional, human, legal, technological and governance) which influence the success of implementation, adoption, use, sustainability and scaling-up of telemedicine. Some of these dimensions have been precisely identified in this exploratory work. Our findings also suggest that more research and evaluation, based on these theoretical and conceptual frameworks, should be suggested from a macro-analytical and holistic perspective in order to fully understand the multidimensional and interdependent factors that influence the scaling-up of telemedicine in Norway.

### Strengths and limitations

The strength of this work, in continuity with previous ones [2, 14], is to provide a global picture of the use of telemedicine over a period of 7 years at regional, organizational and clinical level in Norway. Such systemic and long-term studies on statewide adoption of telemedicine are still lacking in the literature. In addition, the current study identified a number of multilevel factors that could explain the current level of use as well as the challenges to achieve sustainability and scaling-up of telemedicine in Norway.

We recognize some limitations to this study. The quantitative data are related to use of videoconferencing only. As a consequence, other types of telemedicine (e.g. store-and-forward) were not covered. However, this study was based on a unique national database with high-quality and accurate data. We also acknowledge the limited number of interviews. However, saturation was reached with the stakeholders included in this study, also with regard to the rich and diversified documentation which was analyzed. Moreover, multiple perspectives from interviewees who had a thorough knowledge of the issues of telemedicine in Norway were sought to provide an overall understanding of the factors affecting adoption and use.

## Conclusions

The present paper reports unique statewide data on the adoption and utilization of telemedicine in Norway. Despite a very low level of utilization, not exceeding 0.1% of the total outpatient activity, telemedicine was used in the services provision in all 4 regions, 24 hospitals (out of 29, excluding the private sector) and 29 (out of 45) different clinical specialties between 2009 and 2015. Some of these experiences have become routine services. These data shows that telemedicine is considered as a relevant solution at all healthcare levels. However, utilization remains overall low and fragmented across hospitals and clinical specialties. As a consequence, exploitation of the full potential of telemedicine compared with the existing need is still far from being reached.

Moreover, this study provides new insights on a number of conditions for successful integration of telemedicine into health systems, including governance and strategy, organizational and professional dimensions, and economic and financial dimensions. The complexity of such dimensions should be taken into account in future research and evaluation of telemedicine and e-health. In addition, the study strengthens the importance of having a global, inclusive, multi-stakeholders (professional, technological, organizational, political and citizens) strategy that considers telemedicine from an integrated and synergic professional, clinical, organizational, technological and systemic development perspective. This requires the establishment of co-construction and co-evolution approaches, adapted governance, coordinated and coherent funding mechanisms as well as adapted change management. These changes accompany various transformations, often slow, complex and of non-linear nature, that affect both cultures (professional and organizational) and models of production and delivery of services.

The identification of the main implementation and utilization challenges might support decision makers and practitioners in the successful scaling-up of telemedicine. In addition, this work provides a knowledge base useful to other countries which intend to implement telemedicine or other digital health services into their healthcare systems.

## Additional files

Additional file 1: Interview guide. (DOCX 14 kb)

Additional file 2: Outpatient visits and telemedicine consultations in the period 2009–2015 in the different clinical specialties. (DOCX 19 kb)

## Abbreviations

EHR: Electronic health record
GP: General practitioner
ICTs: Information and communications technologies
NPR: Norwegian Patient Registry
