# Supplementary material for: Exploring factors associated with the uneven utilization of telemedicine in Norway: a mixed methods study
Source: BMC Med Inform Decis Mak. 2017 Dec 28;17:180. doi: 10.1186/s12911-017-0576-4 (PMC5745591; doi:10.1186/s12911-017-0576-4)
Supplement: Supplementary file 1 — Interview guide. (DOCX 14 kb) [file 12911_2017_576_MOESM1_ESM.docx]

Additional file 1 Interview guide

| **Organizational level (Hospital)** | **Regional and central levels** |
| --- | --- |
| 1. Does your hospital have a telemedicine strategy? 2. Is telemedicine included in the hospital services organization plan? 3. Are there guidelines for use telemedicine (e.g. procedures, quality monitoring, and evaluation)? 4. Are there service contracts signed between the organizations participating in the delivery of telemedicine?    1. *If yes, do requests from other organizations have the same priority than those from inside the organization? Or are physicians required to respond to all internal requests before responding to other external requests?*    2. *Are doctors obliged to provide telemedicine services, or they may refuse to use telemedicine (autonomy of practice)?* 5. How does telemedicine impact on the plan of medical staff in hospitals (recruitment, retention, etc.)?    1. *How turnover of staff may impact on delivery and long-term use of telemedicine?*    2. *Do some (small) hospitals refuse to use telemedicine for fear of losing medical staff?* 6. Are there strategies for communication and promotion of telemedicine to healthcare professionals? 7. Are there any formal change management strategies? 8. Is there any clinical and organizational leadership that allow legitimizing telemedicine?   **Economic and financial aspects**   1. Which are the financial mechanisms for telemedicine services in hospitals? 2. How does use of telemedicine influence allocation of budget in the hospital? 3. Are there additional support mechanisms for hospital to invest in telemedicine services? 4. How do these financial mechanisms affect the organizations involved (hospital requesting a consultation and hospital providing the consultation)? 5. Who is responsible for the maintenance and the functioning of telemedicine systems (e.g. software, internet connections, update, quality assessment, etc.) and how the responsibilities are shared between hospitals and municipalities, regions, etc.?   **Evolution of telemedicine activity**   1. Do you have an idea about the evolution of telemedicine activity in your organization and region, especially just after 2009 and 2013? (Show the curve of evolution of the use over the same period in the hospital and the region) 2. Do you have an idea about the evolution (increased and decreased activity) in the country, especially just after 2009 and 2013? (Show the curve of evolution of the use over the same period in the country) | 1. Are there national and/or regional digital health plans? 2. Are there national and/or regional strategies specifically focused on telemedicine?    1. *If yes, is there coordination between telemedicine strategies and other digital health plans?* 3. Is there a coordination of strategies and investments between different healthcare levels, including the regions, municipalities and hospitals?    1. *How this coordination could support a better use and diffusion of telemedicine?* 4. How telemedicine responsibilities are shared between these levels? 5. Are there national and/or regional strategies for communication and promotion of telemedicine to managers, healthcare professionals, or even the population? 6. How does telemedicine influence regional planning services and staffing plans (e.g. posts allocation for doctors, posts transfers, etc.)? 7. Is there any systematic evaluation of the implemented telemedicine services and/or projects? (*If yes, how lessons learned are shared?)*   **Economic and financial aspects**   1. How is telemedicine financed at national and regional level? (It is enough? It is coordinated?) 2. How does use of telemedicine influence allocation of budget (regional and organizational)? 3. Are there additional support mechanisms for regions or hospitals to invest in telemedicine services? 4. Who is responsible for the maintenance and the functioning of telemedicine systems (e.g. software, internet connections, update, quality assessment, etc.) and how the responsibilities are shared between hospitals and municipalities, regions, etc.? 5. Which are the financial mechanisms for telemedicine services between public and private hospitals/specialists?   **Evolution of telemedicine activity**   1. Do you have an idea about the evolution of telemedicine activity in your region, especially just after 2009 and 2013? (Show the curve of evolution of the use over the same period in the hospital and the region) 2. Do you have an idea about the evolution (increased and decreased activity) in the country, especially just after 2009 and 2013? (Show the curve of evolution of the use over the same period in the country)   **Others**   1. Why do some large hospitals (e.g. Oslo) hardly provide telemedicine services (despite specialties and expertise are often concentrated in university hospitals)? 2. What is the position of the Norwegian Medical Association on the use of telemedicine? Does it encourage (or discourage) the use of telemedicine by doctors? |
