# Supplementary material for: Exploring factors associated with the uneven utilization of telemedicine in Norway: a mixed methods study
Source: BMC Med Inform Decis Mak. 2017 Dec 28;17:180. doi: 10.1186/s12911-017-0576-4 (PMC5745591; doi:10.1186/s12911-017-0576-4)
Supplement: Supplementary file 2 — Outpatient visits and telemedicine consultations in the period 2009–2015 in the different clinical specialties. (DOCX 19 kb) [file 12911_2017_576_MOESM2_ESM.docx]

**Additional file 2.** Outpatient visits and telemedicine consultations in the period 2009-2015 in the different clinical specialties.

| Clinical specialty | Outpatient visits (2009) | Outpatient visits (2010) | Outpatient visits (2011) | Outpatient visits (2012) | Outpatient visits (2013) | Outpatient visits (2014) | Outpatient visits (2015) | Telemedicine contacts (2009) | Telemedicine contacts (2010) | Telemedicine contacts (2011) | Telemedicine contacts (2012) | Telemedicine contacts (2013) | Telemedicine contacts (2014) | Telemedicine contacts (2015) |
| --- | --- | --- | --- | --- | --- | --- | --- | --- | --- | --- | --- | --- | --- | --- |
| Skin and venereal diseases | 197,707 | 204,552 | 189,415 | 222,658 | 216,227 | 221,708 | 244,832 | 220 (0.11%) | 239 (0.12%) | 93 (0.05%) | 9 (0.00%) | 5 (0.00%) | 140 (0.06%) | 679 (0.28%) |
| Rehabilitation | 195,457 | 207,436 | 222,851 | 237,551 | 235,241 | 229,878 | 239,274 | 389 (0.20%) | 162 (0.08%) | 789 (0.35%) | 1,719 (0.72%) | 1,853 (0.79%) | 1,050 (0.46%) | 289 (0.12%) |
| Eye diseases | 228,680 | 266,363 | 287,130 | 301,316 | 311,324 | 327,870 | 347,050 | 154 (0.07%) | 137 (0.05%) | 231 (0.08%) | 229 (0.08%) | 291 (0.09%) | 198 (0.06%) | 20 (0.01%) |
| Obstetrics | 482,661 | 502,400 | 513,143 | 508,829 | 510,189 | 525,568 | 548,195 | 102 (0.02%) | 18 (0.00%) | 14 (0.00%) | 87 (0.02%) | 71 (0.01%) | 60 (0.01%) | 7 (0.00%) |
| Neurosurgery | 14,701 | 16,858 | 19,144 | 20,401 | 21,037 | 22,781 | 21,417 | 803 (5.46%) | 384 (2.28%) | 469 (2.45%) | 274 (1.34%) | 469 (2.23%) | 302 (1.33%) | 4 (0.02%) |
| Orthopedic surgery | 647,839 | 708,595 | 739,050 | 744,042 | 782,432 | 797,401 | 824,641 | 60 (0.01%) | 72 (0.01%) | 76 (0.01%) | 139 (0.02%) | 23 (0.00%) | 23 (0.00%) | 4 (0.00%) |
| Urology | 135,833 | 148,557 | 160,630 | 166,324 | 170,295 | 171,210 | 178,832 | 49 (0.04%) | 9 (0.01%) | 17 (0.01%) | 32 (0.02%) | 15 (0.01%) | 13 (0.01%) | 4 (0.00%) |
| Other clinical specialities (n=8) | 647,404 | 437,965 | 373,906 | 366,197 | 358,986 | 365,725 | 401,748 | 61 (0.01%) | 18 (0.00%) | 2 (0.00%) | 0 (0.00%) | 0 (0.00%) | 1 (0.00%) | 3 (0.00%) |
| Kidney diseases | 53,312 | 52,288 | 54,164 | 57,058 | 58,516 | 218,207 | 224,793 | 12 (0.02%) | 3 (0.01%) | 3 (0.01%) | 11 (0.02%) | 6 (0.01%) | 7 (0.00%) | 2 (0.00%) |
| Pulmonary diseases | 100,842 | 112,729 | 118,963 | 121,942 | 124,625 | 128,164 | 133,082 | 16 (0.02%) | 4 (0.00%) | 20 (0.02%) | 16 (0.01%) | 16 (0.01%) | 11 (0.01%) | 2 (0.00%) |
| Oncology and radiotherapy | 94,416 | 196,456 | 206,934 | 229,263 | 239,773 | 256,901 | 275,288 | 71 (0.08%) | 9 (0.00%) | 2 (0.00%) | 6 (0.00%) | 7 (0.00%) | 10 (0.00%) | 2 (0.00%) |
| Plastic surgery | 52,588 | 55,257 | 59,980 | 57,007 | 63,413 | 72,051 | 75,874 | 0 (0.00%) | 1 (0.00%) | 3 (0.01%) | 0 (0.00%) | 7 (0.01%) | 0 (0.00%) | 1 (0.00%) |
| Haematology | 61,811 | 75,488 | 84,666 | 91,659 | 94,146 | 96,397 | 101,295 | 14 (0.02%) | 0 (0.00%) | 0 (0.00%) | 0 (0.00%) | 0 (0.00%) | 2 (0.00%) | 1 (0.00%) |
| General internal medicine | 68,642 | 65,136 | 61,923 | 53,627 | 55,204 | 59,798 | 67,334 | 29 (0.04%) | 2 (0.00%) | 1 (0.00%) | 0 (0.00%) | 0 (0.00%) | 0 (0.00%) | 1 (0.00%) |
| Cardiovascular surgery | 43,410 | 47,502 | 53,060 | 54,176 | 53,890 | 54,959 | 58,824 | 1 (0.00%) | 1 (0.00%) | 3 (0.01%) | 4 (0.01%) | 0 (0.00%) | 0 (0.00%) | 1 (0.00%) |
| Endocrinology | 108,866 | 117,577 | 119,423 | 125,502 | 130,146 | 132,738 | 132,837 | 16 (0.01%) | 2 (0.00%) | 5 (0.00%) | 23 (0.02%) | 26 (0.02%) | 13 (0.01%) | 1 (0.00%) |
| Pregnancy and parathyroid surgery | 2,171 | 9,883 | 15,717 | 17,186 | 21,182 | 32,414 | 46,193 | 0 (0.00%) | 0 (0.00%) | 0 (0.00%) | 0 (0.00%) | 0 (0.00%) | 0 (0.00%) | 0 (0.00%) |
| Digestive diseases | 122,480 | 142,525 | 162,385 | 174,526 | 175,016 | 178,328 | 193,016 | 23 (0.02%) | 1 (0.00%) | 11 (0.01%) | 31 (0.02%) | 23 (0.01%) | 17 (0.01%) | 0 (0.00%) |
| Rheumatology | 117,566 | 138,881 | 155,656 | 163,420 | 165,226 | 178,662 | 188,859 | 2 (0.00%) | 1 (0.00%) | 0 (0.00%) | 1 (0.00%) | 0 (0.00%) | 0 (0.00%) | 0 (0.00%) |
| Neurology | 143,640 | 150,588 | 184,850 | 170,295 | 170,908 | 181,819 | 188,303 | 369 (0.26%) | 12 (0.01%) | 20 (0.01%) | 32 (0.02%) | 12 (0.01%) | 11 (0.01%) | 0 (0.00%) |
| Anesthesiology | 41,515 | 44,411 | 36,567 | 47,811 | 47,342 | 50,589 | 50,539 | 2 (0.00%) | 3 (0.01%) | 1 (0.00%) | 0 (0.00%) | 0 (0.00%) | 0 (0.00%) | 0 (0.00%) |
| General surgery | 166,569 | 146,575 | 133,809 | 128,941 | 120,962 | 122,353 | 114,238 | 35 (0.02%) | 10 (0.01%) | 7 (0.01%) | 16 (0.01%) | 4 (0.00%) | 5 (0.00%) | 0 (0.00%) |
| Cardiovascular diseases | 222,183 | 228,739 | 249,516 | 260,106 | 263,619 | 269,139 | 267,691 | 152 (0.07%) | 58 (0.03%) | 51 (0.02%) | 30 (0.01%) | 42 (0.02%) | 29 (0.01%) | 0 (0.00%) |
| Children’s diseases | 212,285 | 215,727 | 198,207 | 219,036 | 218,822 | 237,144 | 252,465 | 120 (0.06%) | 25 (0.01%) | 5 (0.00%) | 11 (0.01%) | 7 (0.00%) | 3 (0.00%) | 0 (0.00%) |
| Ear, nose and throat diseases | 324,964 | 333,776 | 353,326 | 356,886 | 365,251 | 382,869 | 403,380 | 15 (0.00%) | 9 (0.00%) | 3 (0.00%) | 2 (0.00%) | 0 (0.00%) | 1 (0.00%) | 0 (0.00%) |
| Gastroenterological surgery | 114,129 | 123,111 | 126,741 | 130,506 | 141,967 | 151,661 | 162,777 | 9 (0.01%) | 4 (0.00%) | 1 (0.00%) | 25 (0.02%) | 2 (0.00%) | 2 (0.00%) | 0 (0.00%) |
| Maxillofacial and mouth diseases | 29,634 | 27,554 | 26,746 | 28,005 | 29,006 | 26,974 | 28,477 | 10 (0.03%) | 2 (0.01%) | 0 (0.00%) | 0 (0.00%) | 0 (0.00%) | 0 (0.00%) | 0 (0.00%) |
| Geriatrics | 16,338 | 16,931 | 18,427 | 19,370 | 18,797 | 17,841 | 18,428 | 10 (0.06%) | 0 (0.00%) | 0 (0.00%) | 2 (0.01%) | 0 (0.00%) | 0 (0.00%) | 0 (0.00%) |
| Infectious diseases | 28,603 | 31,889 | 34,297 | 38,322 | 39,646 | 41,353 | 43,099 | 1 (0.00%) | 0 (0.00%) | 0 (0.00%) | 1 (0.00%) | 0 (0.00%) | 0 (0.00%) | 0 (0.00%) |
| **Total** | **4,676,246** | **4,825,749** | **4,960,626** | **5,111,962** | **5,203,188** | **5,552,502** | **5,832,781** | **2,745 (0.06%)** | **1,186 (0.02%)** | **1,827 (0.04%)** | **2,700 (0.05%)** | **2,879 (0.06%)** | **1,898 (0.03%)** | **1,021 (0.02%)** |
